# Sheng-Jiang powder ameliorates NAFLD via regulating intestinal microbiota in mice

**DOI:** 10.3389/fmicb.2024.1387401

**Published:** 2024-05-27

**Authors:** Pengcheng Zhang, Juan Li, Yifan Miao, Xianlin Zhao, Lv Zhu, Jiaqi Yao, Meihua Wan, Wenfu Tang

**Affiliations:** ^1^Regenerative Medicine Research Center, Sichuan University West China Hospital, Chengdu, China; ^2^Department of Integrated Traditional Chinese and Western Medicine, West China Hospital, Sichuan University, Chengdu, China; ^3^Department of Emergency Medicine, Hospital of Chengdu University of Traditional Chinese Medicine, School of Clinical Medicine, Chengdu University of Traditional Chinese Medicine, Chengdu, China

**Keywords:** NAFLD, traditional Chinese medicine, Sheng-Jiang powder, intestinal microbiota, 16S rDNA

## Abstract

**Background:**

Intestinal microbiota have been demonstrated to be involved in the development of NAFLD, while the relationship between the severity of NAFLD and intestinal microbiota is still not fully elucidated. Sheng-Jiang Powder (SJP) showed exact efficacy in treating SFL and great potential in regulating intestinal microbiota, but the effects need to be further addressed in NASH and liver fibrosis.

**Objectives:**

To investigate the differences in intestinal microbiota of NAFLD with different severity and the effect of SJP on liver damage and intestinal microbiota.

**Design:**

NAFLD mice models with different severity were induced by high-fat diet (HFD) or choline-deficient, L-amino acid-defined high-fat diet (CDAHFD) feeding and then treated with SJP/normal saline.

**Methods:**

Biochemical blood tests, H&E/Masson/Oil Red O/IHC staining, Western blot, and 16SrDNA sequencing were performed to explore intestinal microbiota alteration in different NAFLD models and the effect of SJP on liver damage and intestinal microbiota.

**Results:**

Intestinal microbiota alteration was detected in all NAFLD mice. SJP induced increased expression of Pparγ and alleviated liver lipid deposition in all NAFLD mice. Microbiome analysis revealed obvious changes in intestinal microbiota composition, while SJP significantly elevated the relative abundance of *Roseburia* and *Akkermansia*, which were demonstrated to be beneficial for improving inflammation and intestinal barrier function.

**Conclusion:**

Our results demonstrated that SJP was effective in improving lipid metabolism in NAFLD mice, especially in mice with SFL. The potential mechanism may be associated with the regulation of intestinal microbiota.

## 1 Introduction

Nonalcoholic fatty liver disease (NAFLD) covers different degrees of metabolic abnormalities, from simple steatosis (SFL) to steatohepatitis (NASH), liver fibrosis (NLF) and finally cirrhosis, which is the main cause of end-stage liver disease and hepatocellular carcinoma (Higarza et al., 2021). Nowadays, the prevalence rate of NAFLD is 25.24% worldwide (Lin et al., 2021), and the incidence rate is rising year over year along with the prevalence of obesity globally, and is predicted to reach 100.9 million in 2030 (Estes et al., 2018). Thus, NAFLD causes a huge social disease and economic burden to humans.

In recent years, more and more evidence has shown that intestinal microbiota are the source of hepatotoxic oxidative damage (Borrelli et al., 2018), and they play an important role in the occurrence and development of NAFLD (Liu et al., 2019). After Le Roy et al. first confirmed the decisive role of intestinal microbiota on the development of NAFLD through intestinal microbiota transplantation (Le Roy et al., 2013), an increasing number of studies have found that the alteration of intestinal microbiota promotes the occurrence and development of NAFLD by affecting liver gene expression, energy expenditure, lipid metabolism, insulin resistance and cytokine production. For example, altered intestinal microbiota affects the synthesis of triglycerides and the metabolism of fatty acids, increases toxic lipid metabolites, and then affects mitochondrial function and the oxidative stress response, and ultimately promotes the occurrence of liver inflammation (Leung et al., 2016). Then, altered intestinal microbiota leads to increase of intestinal mucosal permeability and bacterial translocation, which will aggravate the development of liver inflammation (Li et al., 2022). Moreover, NAFLD patients have an increased abundance of gram-negative bacteria in the gut microbiota, which means there are more lipopolysaccharides released into blood from the outer membrane after the death of these bacteria, and endotoxemia will promote inflammatory cytokines release and inflammatory pathway activation, and then promote the development of NAFLD (Khan et al., 2019; Fei et al., 2020). Also, altered intestinal microbiota can regulate bile acid metabolism and inhibit the farnesol-like X receptor (FXR) signaling pathway to promote the progression of NAFLD (Jiao et al., 2018). Additionally, altered intestinal microbiota increases the synthesis of endogenous ethanol, disrupts intestinal tight junctions and increase intestinal mucosal permeability, where endotoxin and ethanol cause direct damage to the liver (Yang et al., 2019; Huang et al., 2021). It is worth noting that intestinal microbiota and NAFLD may interact with each other. On the one hand, intestinal microbiota determine the occurrence and development of NAFLD (de Minicis et al., 2014), and on the other hand, the occurrence of NAFLD can also lead to changes in the intestinal microbiota of mice and humans (Henao-Mejia et al., 2012). However, it is unclear how different stages of NAFLD affect the composition of the intestinal microbiota.

NAFLD currently lacks definitive therapeutic agents (Loomba and Sanyal, 2013). However, based on current research, antioxidants, antidiabetic drugs, and lipid-lowering agents have demonstrated certain therapeutic effects on some patients with fatty livers (Cossiga et al., 2019). With further investigation, targeting the gut microbial composition with prebiotics, probiotics, and synbiotics has introduced a new therapeutic strategy for NAFLD (Safari and Gérard, 2019). Nevertheless, it should be noted that the aforementioned medications primarily target specific aspects of the disease, highlighting the need for a comprehensive drug that addresses the multifactorial nature of NAFLD.

Sheng-Jiang Powder (SJP) is a classical Traditional Chinese Medicine (TCM) prescription for the treatment of liver diseases (Chen et al., 2017). It contains four natural medicinal ingredients: Rhubarb (*Rheum officinale* Baill.), Curcuma (*Curcuma longa* L.), Stiff silkworm and Cicada slough. This prescription is derived from Ten Thousand Diseases Reviving Spring written by Gong Tingxian in the Ming Dynasty of China and has been used clinically for more than 400 years (Chen et al., 2017). Modern pharmacological studies have shown that SJP has good lipid reduction, insulin resistance improvement and anti-inflammatory effects (Miao et al., 2018). Our present studies have demonstrated that SJP significantly inhibited the body weight gain and attenuated liver pathological damage in mice with SFL induced by high-fat diet feeding, and potential mechanism may be related to the regulation of lipid metabolism related signal pathways and intestinal microbiota (Li et al., 2018). Thus, SJP has shown comprehensive effects that can target the complex physiopathology of NAFLD. However, our present results were limited in SFL mice. Whether SJP can regulate the lipid metabolism and intestinal microbiota and further attenuate liver damage in mice with NASH or NLF needs to be further verified.

Therefore, the purpose of this study was to investigate the effects of SJP on liver injury and intestinal microbiota in mice with varying severity of NAFLD, so as to clarify possible protective mechanisms for NAFLD and provide better evidence for clinical application.

## 2 Materials and methods

### 2.1 Ethics statement

The Ethics Committee for Animal Experiments of Sichuan University approved the protocol (Ethics approval number 2017052A). Following the guidelines established by the European Communities Council Directive on November 24, 1986 (86/609/EEC), animal experiments were conducted. It was ensured that all necessary steps were taken to minimize both the number of animals used and their suffering.

### 2.2 Animals and treatment

Male C57BL/6 mice weighing 18–20 g were provided by Charles River (Beijing, China). All mice were fed at controlled temperature (22–23°C) and 12 h light/dark cycle, and then NAFLD was induced by high-fat diet (HFD) or choline-deficient, L-amino acid-defined high-fat diet (CDAHFD). HFD (TP23300: 19% protein, 21% carbohydrate, 60% fat, and 5.1 kcal/g heat density) and CDAHFD feed (TP36226MCD/G: choline deficiency, amino acid-defined high-fat diet (45% fat)) were purchased from Nantong Trophic Animal Feed High-Tech Co., Ltd. (Jiangsu, China). After 1 week of environmental adaptation, the mice were randomly allocated into 9 groups (*n* = 7 per group). The detailed groups were as follows: (1) AC group, fed with control diet for 12 W and normal saline for 6 W; (2) AT group, fed with HFD for 12 W and normal saline for 6 W; (3) AS group, fed with HFD for 12 W and SJP treatment for 6 W; (4) BC group, fed with the control diet CSAA for 6 W and normal saline for 3 W; (5) BT group, fed with CDAHFD for 6 W and normal saline for 3 W; (6) BS group, fed with CDAHFD for 6 W and SJP treatment for 3 W; (7) CC group, fed with CSAA 12 W and normal saline for 6 W; (8) CT group, fed with CDAHFD 12 W and normal saline for 6 W; (9) CS group, fed with CDAHFD 12 W and SJP treatment for 6 W. All mice models received daily intragastric administration of either SJP or an equal volume of saline from the start of the 4th or 7th week. After the collection of fresh feces and blood, mice were immediately euthanized and livers were isolated. The blood and tissue samples were taken for biochemical detection, histopathological analysis, Real-Time PCR analysis and Western blotting.

### 2.3 Preparation of Sheng-Jiang powder

The preparation of SJP has been described in our previous studies (Miao et al., 2019). In short, the composition of SJP used in this experiment was determined according to the dose ratio in *Identification of Typhoid Fever* and is shown in Table 1. Spray drying powder of Rhubarb (batch no. 16110150), Curcuma (batch no. 16080008), Stiff silkworm (batch no. 16100147), and Cicada slough (batch no. 16080020) were purchased from the Affiliated Hospital of Chengdu University of Traditional Chinese Medicine (Chengdu, China). The drug suspension with a concentration of 1 g/mL was prepared with double distilled water. Drug concentrations of 1 g, 5 g, and 10 g SJP/kg BW were tested, and 5 g/kg BW was selected as the optimal dose for treating animals. These doses were selected based on our previous reports (Li and Hu, 2020) and our preliminary experiments. The scientific name of the plant was consulted on January 4, 2023 at https://www.worldfloraonline.org.

**Table 1:** The composition of SJP.

Ingredients	Botanical Latin name	Plant parts	Amount used	Batch No.
Rhubarb	*Rheum officinale Baill.*	Roots and rhizomes	12 g	16,110,150
Curcuma	*Curcuma longa L.*	Rhizome	9 g	16,080,008
Stiff silkworm	NA	NA	6 g	16,100,147
Cicada slough	NA	NA	3 g	16,080,020

### 2.4 Liver histopathological analysis and immunohistochemistry

Mouse liver tissue was fixed in 10% formalin overnight and embedded in paraffin. The liver was then sectioned into 3 μm sections and stained. For IHC, 3% hydrogen peroxide was used to deactivate endogenous peroxidase, while nonspecific signals were blocked with 1% bovine serum albumin. As primary antibody, anti-TGF-β1 (1:150, Abcam, #ab215715) was incubated overnight at 4°C, followed by three washes with PBS buffer and subsequent incubation with a secondary antibody (HRP-Polymer, Biocare Medical) for 60 min at room temperature. The staining process involved the use of 3,3′-diaminobenzidine (DAB) substrate (Biocare Medical) and counterstaining with hematoxylin. All histopathological specimens were reviewed and scored blindly by two independent pathologists, with a scoring system for the scope and severity of tissue injury (0–4 points, steatosis, inflammatory infiltration, necrosis, and fibrosis), as mentioned earlier (Wirtz et al., 2007). The pathological score of liver tissue was the sum of each score.

### 2.5 Oil red O staining

Fat deposition in liver was identified by oil red O staining. The frozen liver tissue was sliced into 5 μm thick sections and subsequently incubated with the oil red O working staining solution for a duration of 20 min. To prepare the staining solution, 0.5 g of Oil Red O was dissolved in 100 mL of isopropyl alcohol to create a stock solution, which was then diluted with distilled water in a ratio of 3:2 to obtain the working solution. Following staining, the sections were rinsed with a buffer solution to eliminate any excess dye, and subsequently re-dyed with hematoxylin.

### 2.6 Serum biomarkers analysis

After fasting for 12 h, blood samples were collected from the inner corner vein and then centrifuged at 2500 rpm for 10 min. The supernatant was taken for biochemical detection. The activity of alanine aminotransferase (ALT), aspartate aminotransferase (AST), serum triglyceride (TG), and total cholesterol (TC) content were determined by Hitachi Automatic Biochemical Analyzer (7170A, Hitachi, Tokyo, Japan) at the Tibet Chengdu Branch of the West China Hospital of Sichuan University, Chengdu, China.

### 2.7 Western blotting analysis

Liver total protein was extracted with RIPA lysis buffer (Cell signaling), followed by sonication. Afterward, extracts were centrifuged for 10 min at 13,000 rpm. Equal amounts of protein were separated by electrophoresis and blotted onto PVDF membrane (Merck Millipore) by wet transfer. After blocking with skimmed milk powder, add the corresponding primary antibody and incubate overnight at 4°C. The following primary antibodies were used: anti-Srebp1c (1:1000, Cell Signaling Technology, #9874), anti-Fasn (1:1000, Cell Signaling Technology, #3189), anti-Pparγ (1:1000, Cell Signaling Technology, #2430), anti-lxr (1:1000, Abcam, #ab176323), anti-TGF-β1 (1:1000, Abcam, #ab215715), and anti-GAPDH (1:3000, Proteintech, 60,004-1-Ig).

### 2.8 Quantitative polymerase chain reaction

The RT-qPCR procedure was described in detail in our previous study (Li and Hu, 2020). At least three independent experiments were used for statistical analysis. Using GAPDH as control, 2^−ΔΔCt^ was used to calculate the gene expression multiple. The primer sequence is shown in Supplementary Table S1.

### 2.9 16SrDNA bioinformatics analysis

The fecal microbiota of mice in each group was detected using the 16SrDNA method. The analysis of fecal samples was conducted by Shenzhen Microecology Technology Co., Ltd. The operation steps in the experiment are carried out in strict accordance with the instructions. The detailed process was reported in our previous work (Li and Hu, 2020).

### 2.10 Statistical analysis

GraphPad Prism (9.0.0) software was used for multiple group comparisons, and one-way ANOVA followed by Fisher’s least significant difference *post hoc* test was used. Two groups were compared using an unpaired Student’s *t*-test (two-tailed). Data are presented as mean ± SEM, with *p* < 0.05 considered statistically significant.

## 3 Results

### 3.1 Construction of various NAFLD mouse models

**Figure 1:**
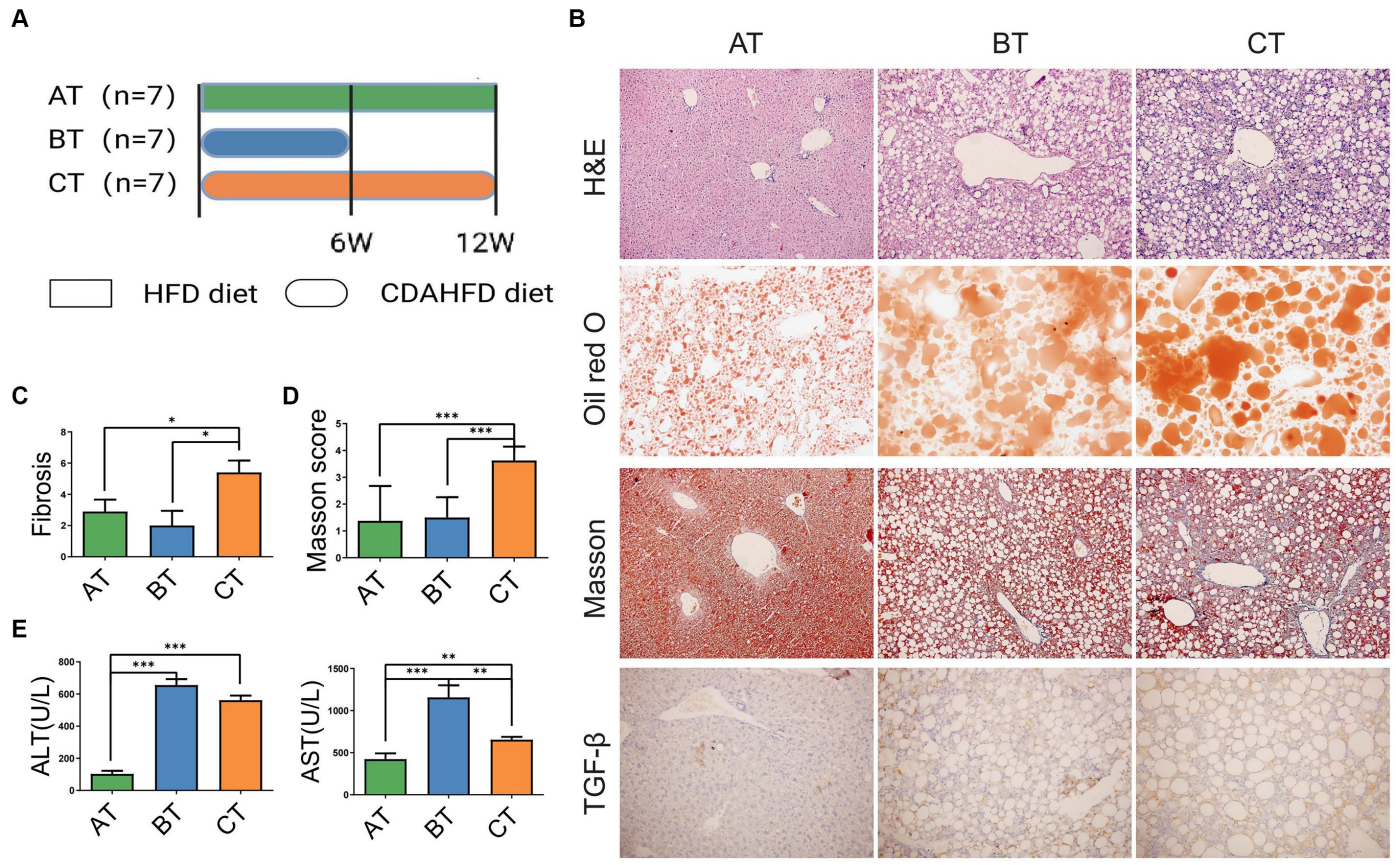
NAFLD mice models with different severity induced by HFD feeding. **(A)** The experimental design and timeline of C57BL/6 mice. **(B)** H&E staining (200×), Oil Red O staining (200×), Masson staining (200×), and IHC staining of liver section (200×). **(C)** Fibrosis scores of the liver tissues in each group. **(D)** Masson scores of the liver tissues in each group. **(E)** Serum ALT and AST levels in each group. Data are expressed as the mean ± SEM. *p < 0.05, **p < 0.01, and ***p < 0.001. AT, simple fatty liver group; BT, non-alcoholic steatohepatitis group; CT, non-alcoholic liver fibrosis group.

We established NAFLD mouse models with different severity to mimic different stages of NAFLD by giving mice three feeding schemes (Figure 1A). At the end of feeding, haematoxylin-eosin (H&E) staining, oil red O staining, Masson staining, and TGF-β were evaluated to confirm the severity of NAFLD according to the related pathological changes (Figure 1B). Notably, we found that liver lipid deposition gradually increased with the severity of fatty liver, the levels of alanine aminotransferase (ALT) and aspartate aminotransferase (AST) significantly increased in the NASH and NLF mice (Figure 1E), and liver fibrosis extent was much severe in NLF group than another two groups according to the H&E staining and Masson scores (*p* < 0.05 and *p* < 0.001, respectively; Figures 1C,D). Thus, the above pathological manifestations of our animal models well simulated the NAFLD with different clinical severity.

### 3.2 Alteration of intestinal microbiota in NAFLD mice models with different severity

#### 3.2.1 Analysis of the intestinal microbial diversity

**Figure 2:**
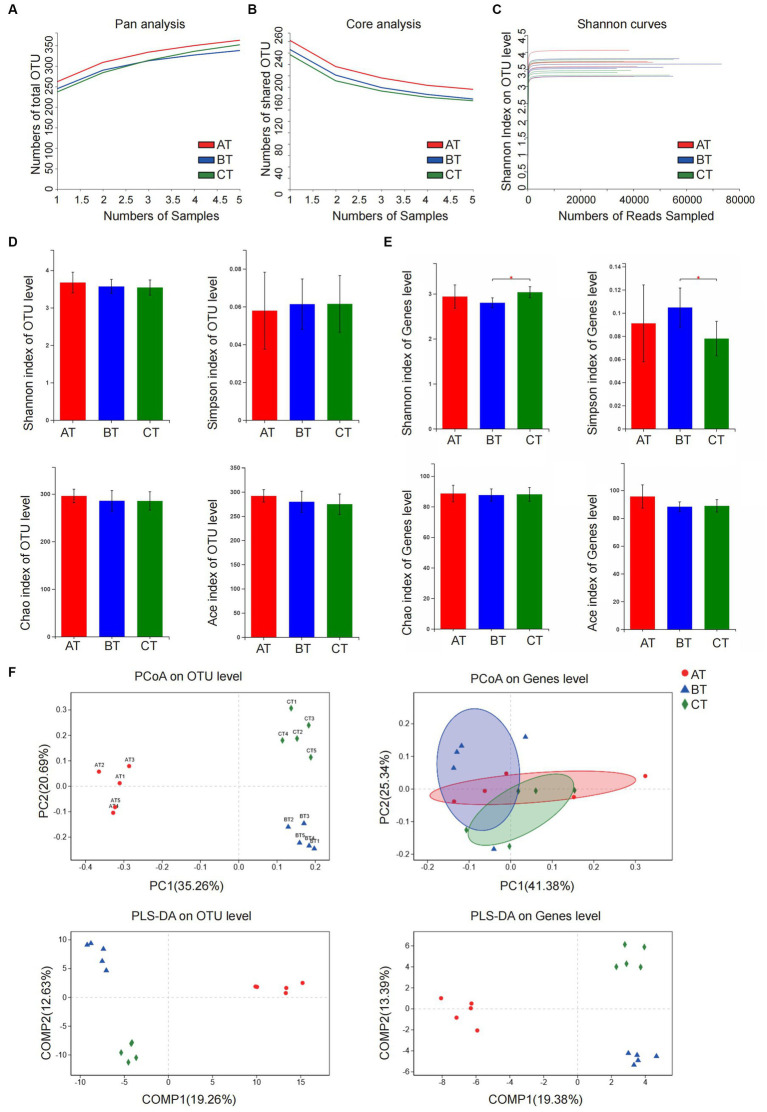
Analysis of microbiota community diversity of intestinal microbiota samples of NAFLD mice after special feeding. **(A)** Pan analysis; **(B)** Core analysis; **(C)** Shannon curves; **(D)** Alpha diversity analysis at OTU level; **(E)** Alpha diversity analysis at the genus level; **(F)** Beta diversity analysis at OTU and genus levels. *p < 0.05 vs. BT group. AT, simple fatty liver group; BT, non-alcoholic steatohepatitis group; CT, non-alcoholic liver fibrosis group.

The results of pan/core analysis showed that the sample size was sufficient to assess the total species richness and the number of core species (Figures 2A,B). The intestinal microbiota diversity was evaluated in the smooth Shannon sparse curve setting, proving that the sequencing depth was sufficient to capture the biodiversity in the test samples (Figure 2C). The α diversity was evaluated by the Shannon index, Simpson index, ACE index, and Chao index. The results showed that the NLF mice decreased in community diversity at the genus level, but there was no significant difference in intestinal community richness among the experimental NAFLD mice with different severity (*p* < 0.05; Figures 2D–E). According to β-diversity analysis of PCoA and PLS-DA, apparent clustering distributions were observed among the three NAFLD groups, suggesting that the microbiota of NAFLD mice with different severity varies and the difference may distinguish different stages of NAFLD (Figure 2F).

#### 3.2.2 Analysis of the intestinal microbial composition

**Figure 3:**
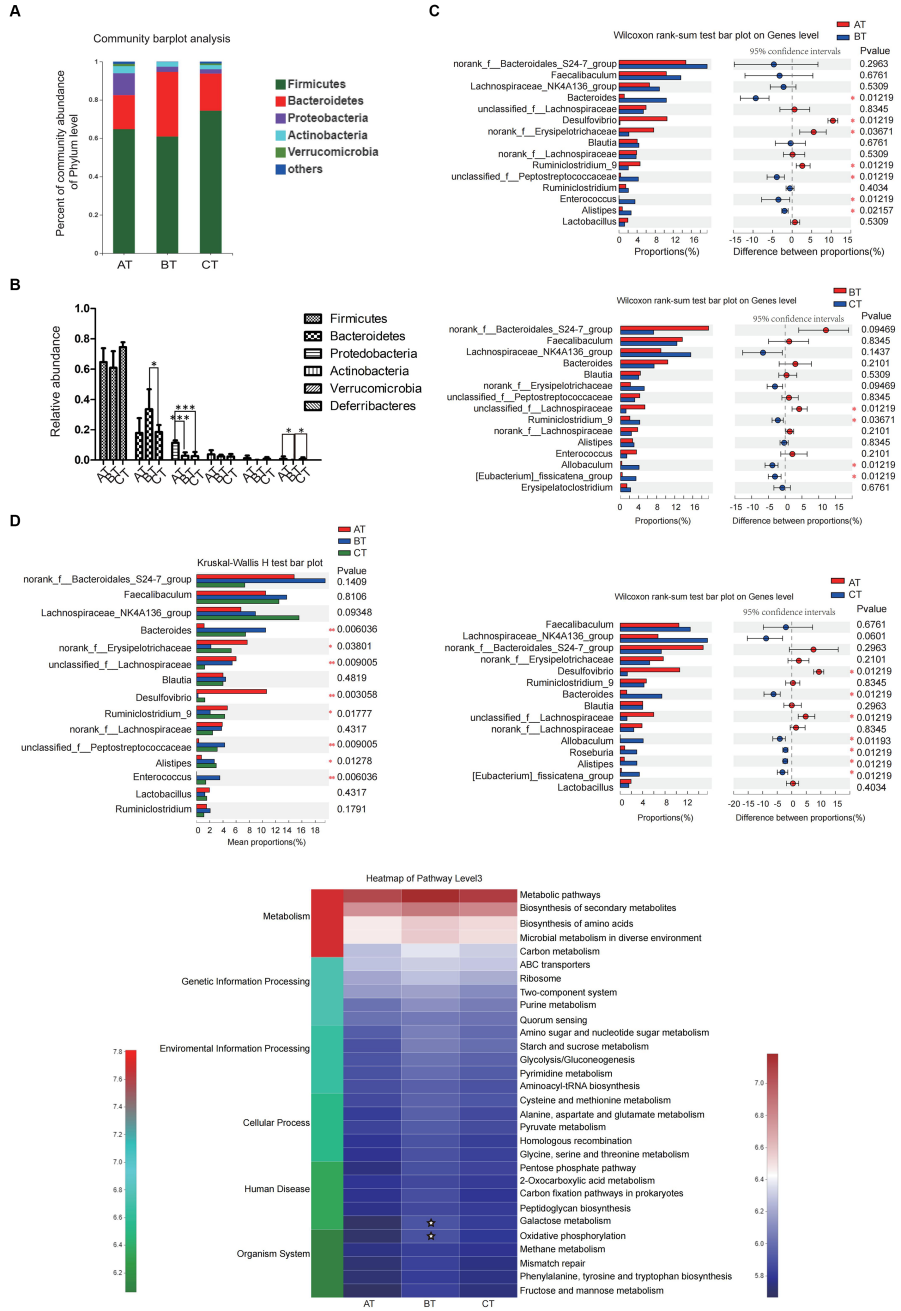
The diversity and relative abundance of intestinal microbiota in NAFLD mice with different severity. **(A)** The relative abundance of intestinal microbiota in each group. **(B)** The relative abundance of intestinal microbiota in each group. **(C)** The between-group microbial community bar plot at the genus level with the top 15 abundant species (AT vs. BT, BT vs. CT, and AT vs. CT). **(D)** PICRUSt2 was used to predict the function of the top 30 abundant species in the intestinal microbiota in each group. Data are expressed as the mean ± SEM. *p < 0.05, **p < 0.01, and ***p < 0.001; ✮*p* < 0.05.

At the phylum level, the intestinal microbiota of each group mainly came from Firmicutes, Bacteroidetes, Verrucomicrobia, Proteobacteria, and Actinobacteria. Compared with the AT group, Proteobacteria in the BT group decreased significantly (*p* < 0.001), and Firmicutes, Actinobacteria, and Verrucomicrobia had a slightly decreasing trend. The ratio of Bacteroidetes of the CT group was significantly lower than that of the BT group (*p* < 0.05), while the ratio of Proteobacteria was significantly lower than that of the AT group (*p* < 0.001; Figure 3A).

At the genus level, there were also significant differences in the abundance of intestinal microbiota in NAFLD mice with different severity. In this experiment, we selected the top 15 high-abundance species in the intestinal microbiota in each group for analysis and comparison. We found that there were significant differences in the abundance of species like Bacteroidetes, norank-f-Erysipelotrichaceae, Desulfovibrio, etc., which contributed to the between-group differences among the three groups that represent different severity of NAFLD (Figure 3B). Differences in the microbial community abundance between groups were further analyzed by Wilcoxon rank-sum test. Our results showed that the abundance of Bacteroidetes and Alistipes in NASH mice increased significantly, while the abundance of Desulfovibrio, norank-f-Erysipelotrichaceae and Ruminiclostridium-9 decreased significantly (all *p* < 0.05) when compared with the SFL mice. And the abundance of unclassified-f-Lachnospiraceae in NLF mice decreased significantly, while the abundance of Ruminiclostridium-9, Allobaculum, and Eubacterium-fissiatena-group increased significantly (all *p* < 0.05) when compared with the NASH mice. Additionally, compared with the SFL mice, the abundance of Desulfovibrio and unclassified-f-Lachnospiraceae in NLF mice was significantly decreased and the abundance of bacteroidetes, Allobaculum, Roseburia, and Alistipes was significantly increased (all *p* < 0.05; Figure 3C).

#### 3.2.3 Function prediction of the intestinal microbiota

In addition, PICRUSt2 was employed to predict the function of the top 30 abundant species in the intestinal microbiota of NAFLD mice with different severity. The heatmap of pathway level3 showed that the enrichment of BT-group mice intestinal microbiota in Galactose metabolism and Oxidative phosphorylation pathways was significantly higher than that of AT group (both *p* < 0.05; Figure 3D), while there was no significant difference in the enrichment of gut microbiota in each pathway level3 between the BT and CT-group mice.

### 3.3 Effect of SJP on HFD-induced SFL

Our previous study (Li and Hu, 2020) has uncovered a series of protective effects of SJP on HFD feeding induced SFL mice, including inhibiting the body weight gain, attenuating lipid accumulation in blood and liver, modulating the expression of Pparγ and ameliorating liver pathological damage. And these effects might be partly attributed to the regulation of gut microbiota as we found that the relative abundance of certain short-chain fatty acids (SCFAs) producing bacteria, such as Roseburia and norank-f-Erysipelotrichaceae were significantly changed after SJP treatment.

### 3.4 Effect of SJP on CDAHFD-induced NASH

#### 3.4.1 SJP attenuated liver inflammatory injury and lipid accumulation

Dose-effect experiments were used to determine the optimal dose of SJP therapy. We observed that doses of 5 g/kg were more effective than doses of 1 g/kg and 10 g/kg (Supplementary Figure S1). Therefore, we chose 5 g/kg as the therapeutic dose for further experiments.

**Figure 4:**
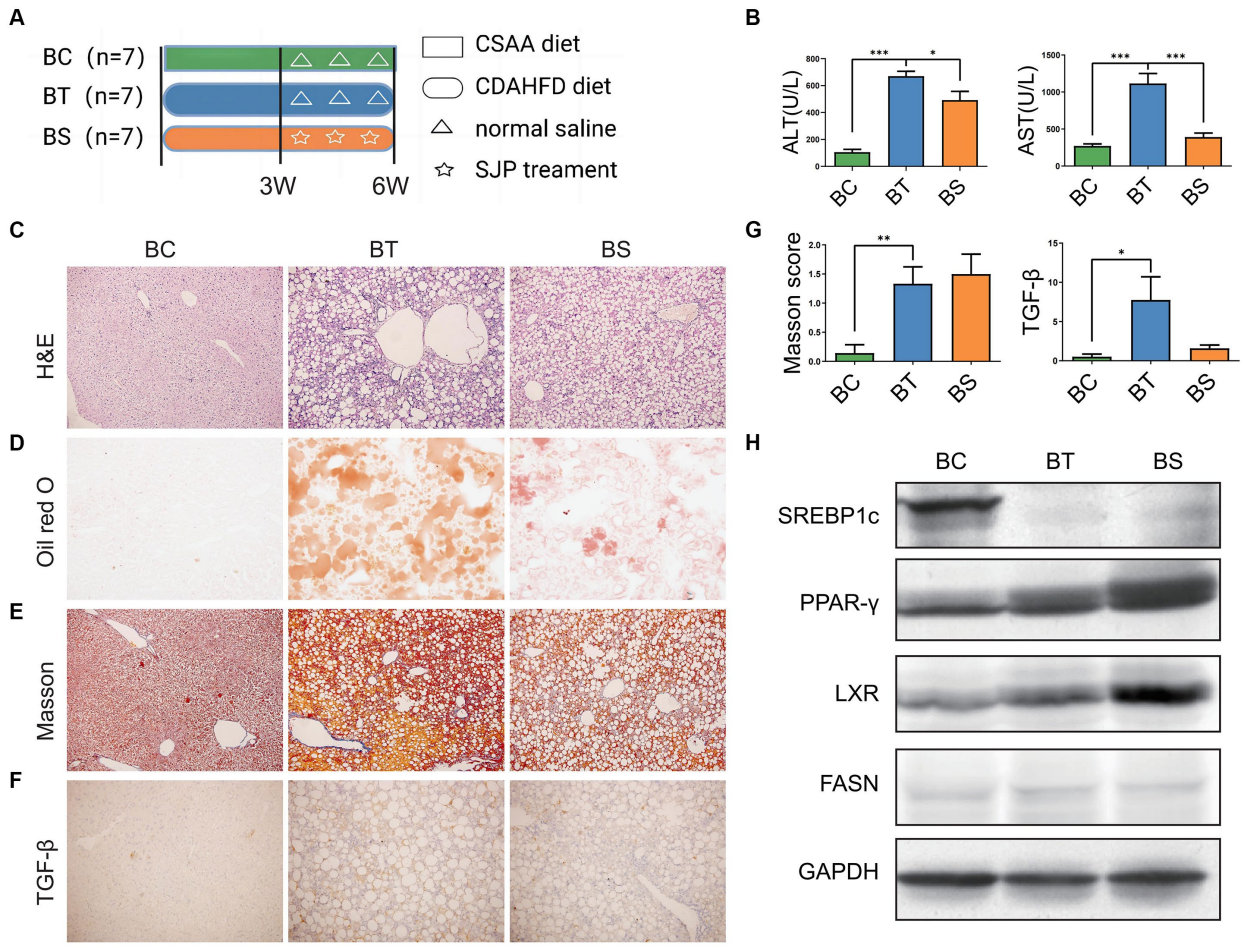
To evaluate the therapeutic effect of SJP on NASH mice. **(A)** The experimental design and timeline of C57BL/6 mice. **(B)** The levels of serum ALT and AST. **(C)** The representative image of H&E staining. **(D)** The representative image of oil red O staining. **(E–G)** The representative image and statistical data of Masson staining and TGF-β IHC staining. **(H)** Western blots were performed to detect protein levels of Lxr, Pparγ, Srebp1c, and Fasn. Magnification, H&E × 200 **(**C**)**, oil red O × 200 **(**D**)**, Masson ×200 **(**E**)**, TGF-β × 200 **(**F**)**. Data are expressed as the mean ± SEM. *p < 0.05, **p < 0.01, and ***p < 0.001. BC, non-alcoholic steatohepatitis control group; BT, non-alcoholic steatohepatitis group; BS, non-alcoholic steatohepatitis + SJP treatment group.

In our experiment, although CDAHFD diet feeding caused weight loss and decreased levels of serum triglyceride (TG) and total cholesterol (TC) in NASH mice (Figure 4A and Supplementary Table S2), it induced apparent elevation of serum ALT and AST, liver lipid accumulation, hepatic steatosis, and inflammatory infiltration and accompanied fibrotic process with an increased TGF-β level and Masson score, which was indeed in accordance with clinical manifestation of NASH. SJP treatment effectively decreased serum ALT and AST levels and ameliorated lipid deposition in liver. But it could not reduce the pathological injury of liver and the subsequent fibrotic changes (Figures 4B–G; Supplementary Figure S2A).

We further detected the protein expression of lipid synthesis related genes (Figure 4H). Western blot analysis revealed that the expression of Srebp1c was significantly downregulated in NASH mice compared with the controls, whereas the expression of Pparγ and Lxr was significantly upregulated (all *p* < 0.05). However, SJP treatment did not reverse the alteration of these lipid synthesis related genes and might even increase Pparγ expression (Supplementary Figure S3A). Taken together, our results showed that SJP could attenuate liver inflammatory injury and lipid deposition in NASH mice.

#### 3.4.2 SJP regulated the intestinal microbiota of NASH mice

##### 3.4.2.1 SJP changed intestinal microbial diversity in NASH mice

**Figure 5:**
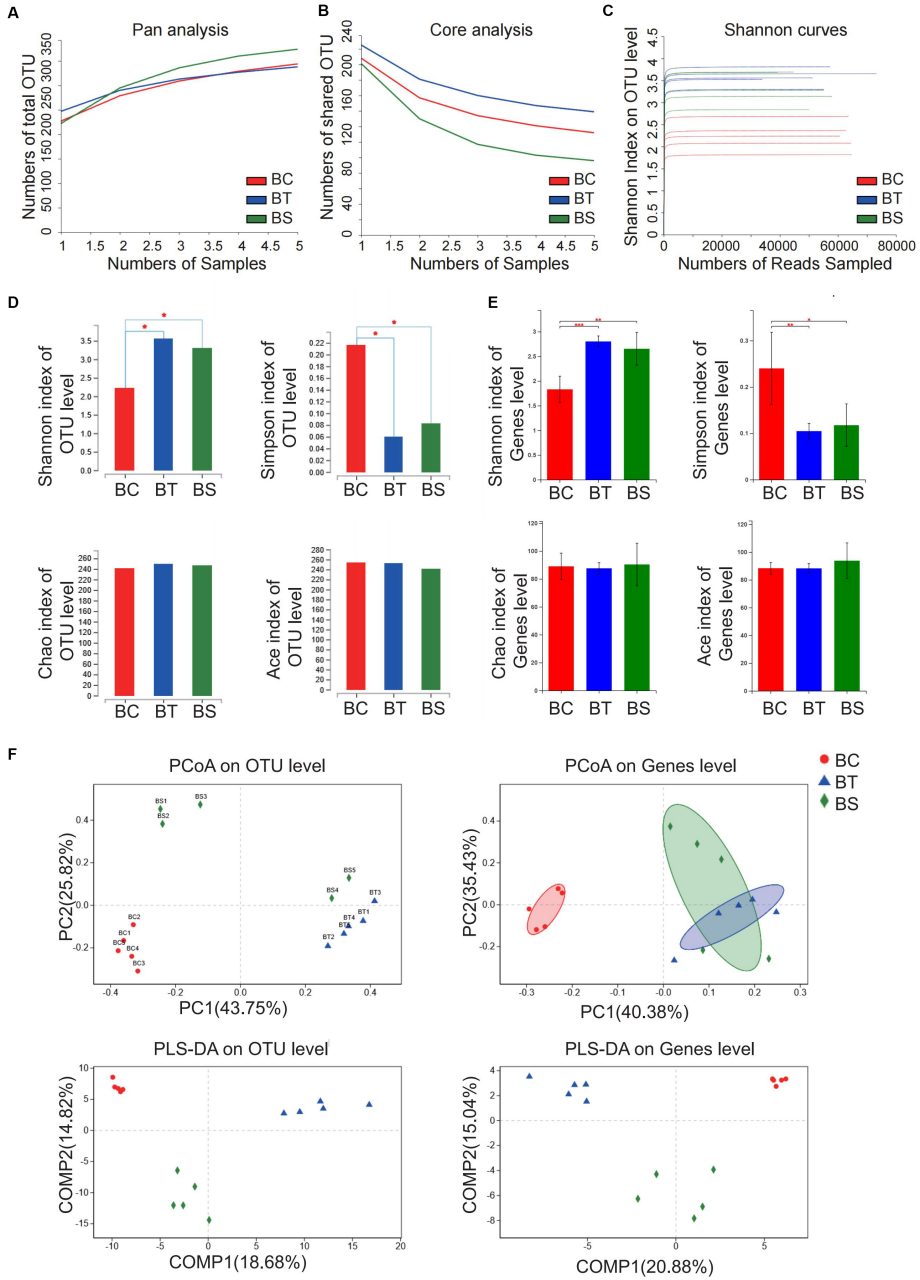
Analysis of microbiota community diversity of intestinal microbiota in NASH mice. **(A)** Pan analysis; **(B)** Core analysis; **(C)** Shannon curves; **(D)** Alpha diversity analysis at the OTU level; **(E)** Alpha diversity analysis at the genus level; **(F)** Beta diversity analysis at OTU and genus levels. *p < 0.05, **p < 0.01, and ***p < 0.001. BC, non-alcoholic steatohepatitis control group; BT, non-alcoholic steatohepatitis group; BS, non-alcoholic steatohepatitis + SJP treatment group.

Pan/core analysis was first conducted to evaluate whether the sample size was sufficient to assess the total species richness and the number of core species, and then the smooth Shannon rarefaction curves were exhibited to demonstrate the sequencing depth was sufficient to capture biodiversity in the tested samples (Figures 5A–C). Alpha diversity analysis indicated an increase in the species of intestinal microbiota community and in the uniformity of species distribution in NASH mice with elevated Shannon and Simpson indexes, while SJP seemed to have very little impact on Alpha diversity (Figures 5D–E). The β-diversity analysis showed apparent clustering distribution among the three groups, which indicated apparent between-group differences in the intestinal microbiota of NASH mice, the controls and the SJP-treated mice (Figure 5F).

##### 3.4.2.2 SJP modulated the intestinal microbiota composition in NASH mice

**Figure 6:**
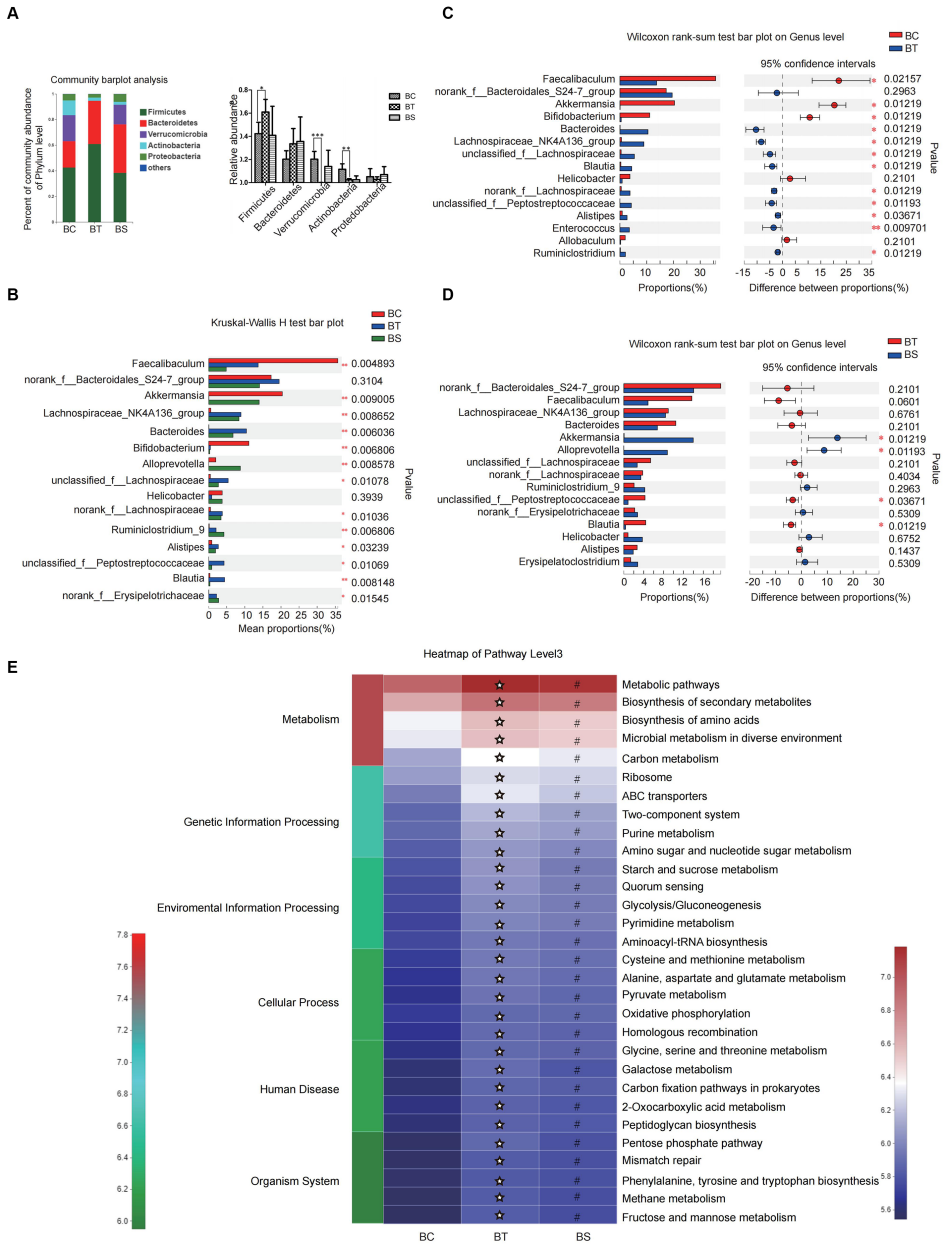
SJP treatment affected the composition of intestinal microbiota in NASH mice. **(A)** The relative abundance of intestinal microbiota in each group. **(B)** The relative abundance of intestinal microbiota in each group. **(C,D)** Microbial community bar plot at the genus level with the relative abundance within the top 15 between groups (BC vs. BT and BT vs. BS). **(E)** PICRUSt2 was used to predict the function of the top 30 abundant species in the intestinal microbiota in each group. Data are expressed as the mean ± SEM. *p < 0.05, **p < 0.01, and ***p < 0.001; ✮*p* < 0.05 vs. BC group; ^#^*p* < 0.05 vs. BC group. BC, non-alcoholic steatohepatitis control group; BT, non-alcoholic steatohepatitis group; BS, non-alcoholic steatohepatitis + SJP treatment group.

At the phylum level, the abundance of Firmicutes in the NASH mice increased significantly, while the abundance of Verrucomicrobia and Actinobacteria decreased significantly when compared with the controls. SJP treatment slightly reversed these changes, but there were no statistical differences (Figure 6A).

At the genus level, we found significant differences in intestinal microbiota among the three groups. Except for Norank-f-Bacteroidales-S24-7 and Helicobacter, the abundance of another 13 top abundant species varied greatly among the three groups, including Akkermansia, Alloprevotella, Norank-f-Erysipelotrichaceae, etc. (Figure 6B). To further identify the species that discriminate NASH from the controls and SJP-treated mice, differences between groups were analyzed by the Wilcoxon rank-sum test. Our results showed that the abundance of Faecalibaculum, Akkermansia and Bifidobacterium in NASH mice was significantly lower than that of controls, while some species were significantly increased, including Bacteroidetes, Blautia, Alistipes, etc. (Figure 6C). SJP treatment significantly increased the abundance of Akkermansia and Alloprevotella and decreased the abundance of Unclassified-f-Peptostreptococeceae and Blautia (Figure 6D). The above results suggested that SJP might have the potential to regulate intestinal dysbiosis in NASH mice.

##### 3.4.2.3 Potential effect of SJP on biological activities *in vivo* from function prediction of the intestinal microbiota

The PICRUSt2 analysis showed that the enrichment of intestinal microbiota in NASH mice was significantly increased in all of the pathway level3 when compared with the controls, while SJP seemed to reverse these changes slightly as we observed a color gradient from red to blue in the heatmap which indicated a gradual decrease in intestinal microbiota enrichment (Figure 6E).

### 3.5 Effect of SJP on CDAHFD-induced NLF

#### 3.5.1 SJP might attenuate liver lipid accumulation in NLF mice

**Figure 7:**
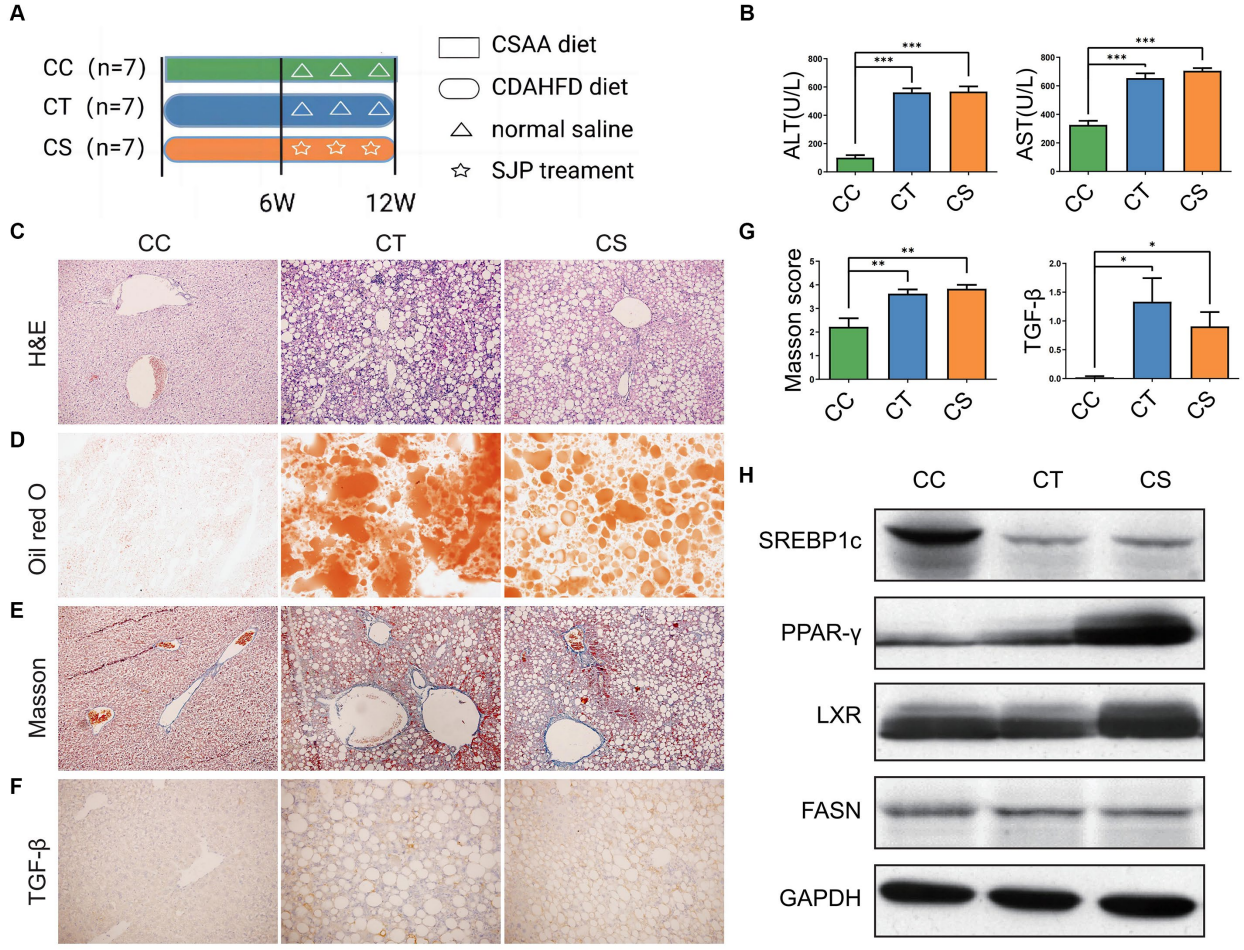
To evaluate the therapeutic effect of SJP on NLF mice. **(A)** The experimental design and timeline of C57BL/6 mice. **(B)** The levels of serum ALT and AST. **(C)** The representative image of H&E staining. **(D)** The representative image of oil red O staining. **(E–G)** The representative image and statistical data of Masson staining and TGF-β immunohistochemical staining. **(H)** Western blots were performed to detect protein levels of Lxr, Pparγ, Srebp1c, and Fasn. Magnification, H&E × 200 **(**C**)**, oil red O × 200 **(**D**)**, Masson ×200 **(**E**)**, TGF-β × 200 **(**F**)**. Data are expressed as the mean ± SEM. *p < 0.05 and ***p < 0.001. CC, liver fibrosis control group; CT, liver fibrosis group; CS, liver fibrosis + SJP treatment group.

Mice were subjected to different treatments according to the experimental design (Figure 7A). As in NASH mice, CDAHFD feeding induced apparent weight loss and serum TG and TC reduction (Supplementary Table S2). But CDAHFD feeding also induced a significant increase in serum ALT and AST, hepatic steatosis and inflammatory infiltration as well as liver fibrosis according to the H&E staining (Figures 7B,C; Supplementary Figure S2B). The oil red O staining suggested significant lipid deposition in the liver, and the Masson score and increased tissue TGF-β expression similarly revealed hepatic fibrosis in NLF mice (Figures 7D–G). SJP treatment significantly altered mRNA and protein expression of lipid-related genes in NLF mice (Figure 7H; Supplementary Figures S3B,C).

#### 3.5.2 SJP regulated the intestinal microbiota of NLF mice

##### 3.5.2.1 SJP changed intestinal microbial diversity in NLF mice

**Figure 8:**
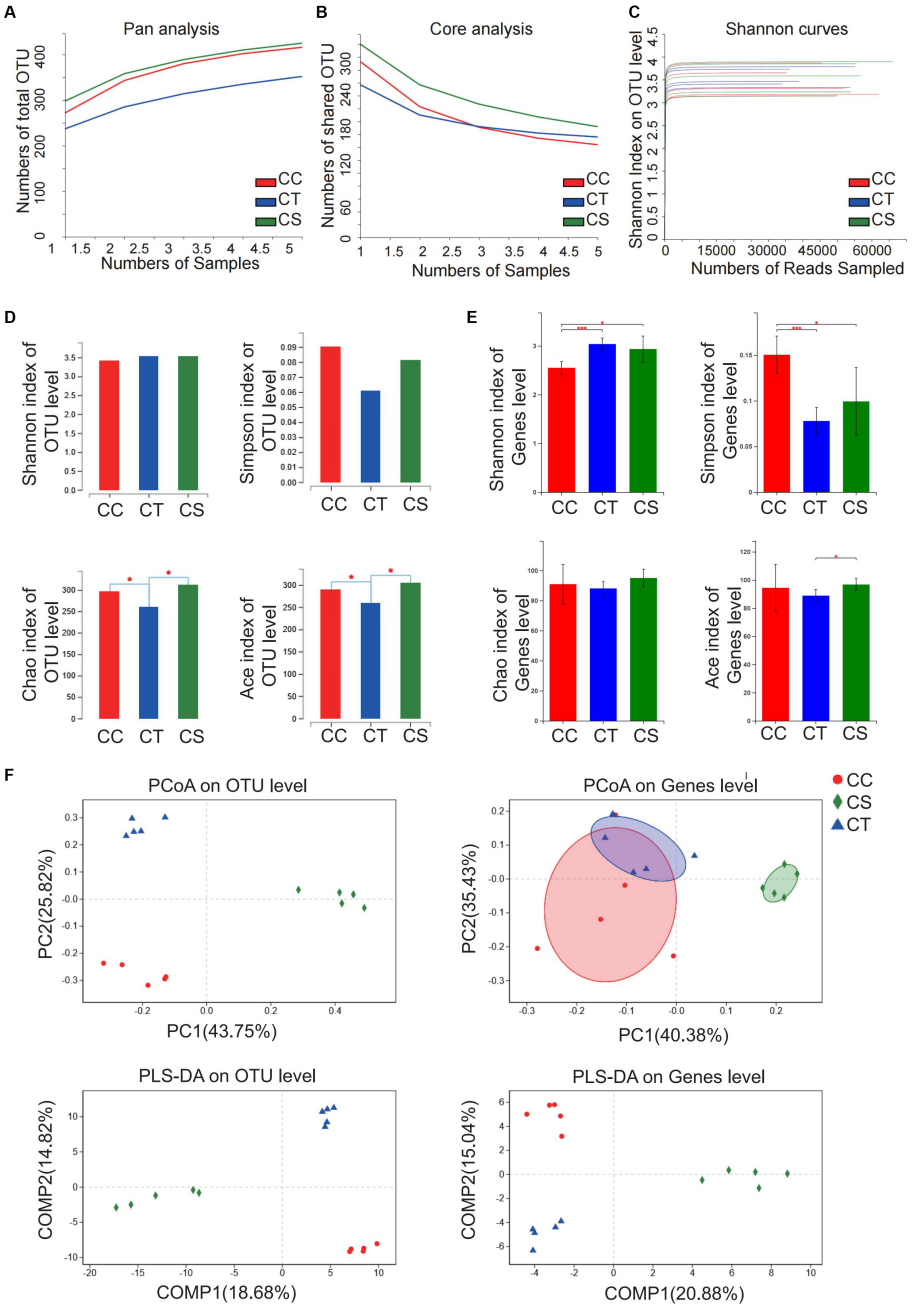
Analysis of microbiota community diversity of intestinal microbiota in NLF mice. **(A)** Pan analysis; **(B)** Core analysis; **(C)** Shannon curves; **(D)** Alpha diversity analysis at OTU level; **(E)** Alpha diversity analysis at the genus level; **(F)** Beta diversity analysis at OTU and genus levels. *p < 0.05 and ***p < 0.001. CC, liver fibrosis control group; CT, liver fibrosis group; CS, liver fibrosis + SJP treatment group.

Similarly, pan/core analysis was first conducted to evaluate whether the sample size was sufficient to assess the total species richness and the number of core species, and then the smooth Shannon rarefaction curves were exhibited to demonstrate the sequencing depth was sufficient to capture biodiversity in the tested samples, and thereafter the intestinal microbial diversity was analyzed (Figures 8A–C). The α diversity analysis revealed an increase in the intestinal microbiota community diversity of NLF mice with elevated Shannon and Simpson indexes at the genus level, but a decrease in the community richness with reduced Chao index and Ace index at the OTU level. SJP treatment significantly increased the intestinal microbiota community richness in NLF mice (Figures 8D–E). Consistently, distinct between-group differences were observed in the intestinal microbiota of NLF mice according to the apparent clustering distribution of individuals from each group, which indicated the alteration of the intestinal microbiota with disease and treatment (Figure 8F).

##### 3.5.2.2 SJP modulated the intestinal microbiota composition in NLF mice

**Figure 9:**
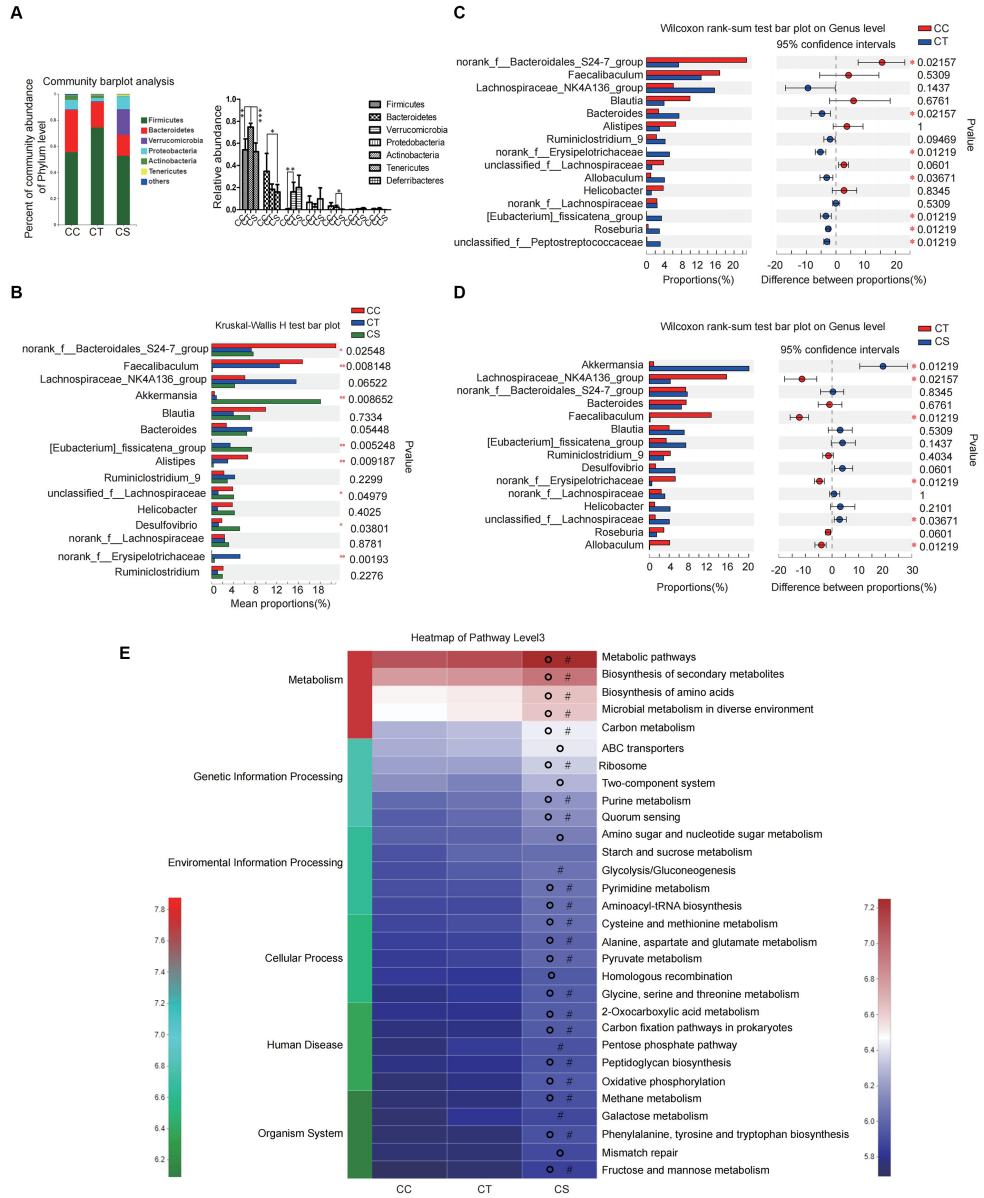
SJP treatment affected the composition of intestinal microbiota in NLF mice. **(A)** The relative abundance of intestinal microbiota in each group. **(B)** The relative abundance of intestinal microbiota in each group. **(C,D)** Microbial community bar plot at the genus level with the relative abundance within the top 15 between groups (CC vs. CT and CT vs. CS). **(E)** PICRUSt2 was used to predict the function of the top 30 abundant species in the intestinal flora of mice. Data are expressed as the mean ± SEM. *p < 0.05, **p < 0.01, and ***p < 0.001; ^#^*p* < 0.05 vs. CC group; ^O^*P* < 0.05 vs. CT group. CC, liver fibrosis control group; CT, liver fibrosis group; CS, liver fibosis + SJP treatment group.

At the phylum level, the abundance of Firmicutes and Verrucomicrobia in NLF mice increased significantly when compared to the controls, while SJP treatment led to a decrease in Firmicutes. Moreover, SJP induced marked decrease in the abundance of Actinobacteria (Figure 9A).

At the genus level, there were significant differences in the abundance of multiple genera among the three groups, including *Akkermansia*, *norank-f-Erysipelotrichaceae*, *Desulfovibrio*, and so on (Figure 9B). Then, further between-group differences analysis showed that the abundance of intestinal *norank-f-Bacteroidales-S24-7-group* decreased significantly in NLF mice, while *Roseburia*, *norank-f-Erysipelotrichaceae*, *Allobaculum*, *Eubacterium-fissiatena-group*, *Unclassified-f-Peptostreptococeceae*, and *Bacteroides* increased significantly (Figure 9C). In addition, the abundance of *Akkermansia* and *unclassified-f-Lachnospiraceae* increased significantly, while the abundance of *norank-f-Erysipelotrichaceae, Lachnospiraceae-NK4A136-group, Faecalibaculum*, and *Allobaculum* decreased significantly after SJP treatment (Figure 9D). The above results suggested that SJP might have the potential to regulate the intestinal microbiota of NLF mice and make them return to a normal state.

##### 3.5.2.3 SJP affects biological processes *in vivo* by regulating the intestinal microbiota in NLF mice

The PICRUSt2 result showed no significant difference in the enrichment of the main intestinal microbiota in pathway level3 between the NLF mice and the controls. However, the enrichment of intestinal microbiota in SJP treated mice was much higher than that of NLF mice. According to our results, the changes in intestinal microbiota caused by SJP affected most of the *in vivo* biological processes, including metabolism, genetic information processing, environmental information processing, cellular process, human disease, and organism system (Figure 9E).

## 4 Discussion

In the present study, we established NAFLD mice models with different severity by special feeding and these models exhibited a series of clinical features that were consistent with different stages of NAFLD. Then, we found that the protective effect of SJP diminished with the NAFLD severity. Briefly, SJP decreased the body weight and serum TG level, regulated the expression of lipid synthesis related genes, improved liver function and hepatic steatosis in SFL mice. SJP reduced the liver enzyme level and hepatic lipid deposition but did not improve liver pathological damage in NASH mice. While in NLF mice, SJP appeared to only affect PPAR γ expression with no obvious protective effect on liver inflammation, steatosis or fibrosis. Despite the protective effect on liver pathology was limited, SJP did reduce hepatic lipid deposition in all NAFLD mice as we observed in oil red O staining. Similarly, we have detected obvious changes in intestinal microbiota in all NAFLD mice after SJP treatment.

Hepatic lipid synthesis is a rigorously regulated biological process that is extremely important for the synthesis of very low-density lipoproteins and tissue energy transfer. Excessive fat intake induces the massive production of triglycerides, the accumulation of lipid droplets in hepatocytes, and ultimately leads to the formation of fatty liver (Wu et al., 2020). The expression of lipid synthesis genes is regulated by a series of transcription factors, including Srebp1c, Lxr, PPARs, etc. (Sohrabi et al., 2020). Srebp1c is the main transcription factor regulating fatty acid synthesis in the liver. Over-expression of Srebp1c can affect lipid accumulation and oxidation in the liver and other non-adipose tissues. When there is excess energy, Srebp1c is activated, which leads to lipid accumulation and ultimately causes fatty liver, insulin resistance, insulin secretion deficiency, and dyslipidemia (Ju et al., 2020). In this experiment, hepatic expression of Srebp1c was significantly reduced in NASH and NLF mice, which seems to be contrary to the current generally increased Srebp1c expression in HFD induced fatty liver models. This may be related to the fact that CDAHFD feeding did not cause obesity in our mice models, as the same decreased Srebp1c expression was also found in the non-obese mouse model of NASH established with MCD feeding by Yamamoto et al. (2016). Pparγ also plays an important role in regulating lipid metabolism, especially in lipid production gene expression and liver lipid synthesis induced by HFD (Liu et al., 2019). Pparγ can improve insulin sensitivity, inhibit lipolysis of adipose tissue, reduce the release of free fatty acids from central adipose tissue, and prevent ectopic fat deposition in the liver and pancreas (Ryyti et al., 2021). Excess energy induces abnormal Pparγ activation in liver and then promotes the occurrence of hepatic fat deposition. In a HFD induced NAFLD animal model, the expression of Pparγ in the liver increased significantly and the liver gradually developed steatosis as time went on (Yu et al., 2003). A high level of Pparγ expression in the liver, however, can prevent fat deposition in other tissues from insulin resistance, while suppressing the expression of other nuclear transcription factors and their downstream inflammatory genes to reduce the inflammatory response, thus realizing self-protection of the body (Gross et al., 2017). In the present study, we found that the hepatic expression of Pparγ tended to increase in all NAFLD mice models, especially in NASH mice, while the expression was further promoted by SJP treatment. The protective effect of SJP on NAFLD might be related to the regulation of Pparγ expression. In addition, Fasn is a fatty acid synthase that is mainly involved in the synthesis of long-chain saturated fatty acids such as palmitic acid, and its expression is regulated by transcription factors such as Srebp1c and Pparγ (Kim et al., 2022). Further studies on the expression of lipid synthesis proteins at different stages of NAFLD may help us better understand how lipid metabolism disorders affect the pathophysiological process of NAFLD.

Intestinal microbiota is a complex ecological community that performs many functions vital to health and survival, including digesting food, producing anti-inflammatory substances, promoting metabolism, and regulating the immune system (Yan et al., 2022). Microbiota affect the physiology and susceptibility to disease of the host through their collective metabolic activities and host interactions. Therefore, the composition of the microbiota at the community level varies with the physiological state of the host and is closely related to the health of the host (Liu et al., 2022). Bacteria are the main members of the intestinal microbiota, and the intestinal microbiota of mice mainly comes from Firmicutes, Bacteroidetes, Verrucomicrobia, Actinobacteria, and Proteobacteria. Among them, Firmicutes and Bacteroidetes constitute the largest components of intestinal microbiota (Duan et al., 2010). Factors such as age, disease, host genes, and dietary habits all affect the composition of the microbiota. Indeed, it is generally believed that diet has a substantial impact on regulating the structure of the intestinal microbiota, which in turn affects intestinal metabolism (Li et al., 2022). Integrative analysis of the microbiota and metabolome of the human intestinal mucosal surface reveals their exquisite interrelationships. Accumulated evidences have revealed that HFD reduced the intestinal microbial diversity and increased Firmicutes/Bacteroidetes flora ratio, thus increased energy acquisition in the gut which provided more substrates for the activation of the lipogenesis pathway while inducing upregulation of genes involved in lipid metabolism in the distal small intestine in both humans and animals (Do et al., 2018). And the idea that increased Firmicutes/Bacteroidetes flora ratio is correlated with the development of NAFLD is well accepted nowadays. Many studies believe that the importance of the Bacteroidetes phylum bacteria in the gut flora is based on their effects on the production of short-chain fatty acids (SCFAs) and the metabolism of the intestinal microbes. For example, a 20% decrease in the fecal Bacteroidetes community and a corresponding increase in Firmicutes bacteria will result in a 150 kcal increase in energy obtained from the diet, while the SCFAs produced in the gut can provide energy to the liver, and they may also reduce energy harvest and protect against fatty liver by increasing Bacteroidetes/Firmicutes ratio (Jumpertz et al., 2011). Our results also detected a significant increase in Firmicutes phylum bacteria in all of the NAFLD mice despite there was no significant changes in the proportion of Bacteroidetes phylum bacteria. In fact, many SCFAs-producing bacteria are from Firmicutes phylum, such as *Roseburia, Blautia, Fusobacterium, Coprococcus, Ruminococcus* and so on. The increased abundance of these bacteria will promote glucose and lipid metabolism, hence benefit for NAFLD.

At the genus level, we found that most of the microorganisms that contributed to the significant differences between groups in NAFLD mice with varying severity had the ability to produce SCFAs, including *Faecalibaculum, Blautia, Lachnospiraceae NK4A136-group, unclassified-f-Lachnospiraceae, Roseburia, norank-f-Lachnospiraceae, Ruminiclostridium-9, Eubacterium_fissicatena-group, norank-f-Erysipelotrichaceae, Lactobacillus, norank-f-Bacteroidales-S24–7-group, Bacteroidales and Allobaculum* et al. SCFAs are the main end products of the bacterial fermentation of soluble dietary fiber and indigestible carbohydrates, mainly including formic acid, acetic acid, propionate and butyric acid (Koh et al., 2016). SCFAs have multiple effects on maintaining human health, such as protecting the intestinal mucosal barrier, acting as the nutritional and energy component of the intestinal epithelium, reducing inflammation, and enhancing gastrointestinal function. They essentially represent the main carbon flow from the intestinal microbiota to the host, changing the energy harvest and regulating metabolic processes, including regulating appetite, promoting energy expenditure, stimulating insulin sensitivity, reducing energy harvest, and thus affecting the development of NAFLD (Morrison and Preston, 2016). It was found that the abundance of intestinal SCFAs-producing bacteria in patients with NAFLD became a characteristic change in gut microbial composition in NASH patients (Rau et al., 2018). Boursier et al. (2016) found that the severity of NAFLD was associated with gut dysbiosis and metabolic functional shifts in the gut microbiota, where increased Bacteroides abundance was independently associated with NASH, and increased Ruminococcus abundance was independently associated with liver fibrosis. In our study, the abundance of *Lactobacillus* and *Bifidobacterium* was significantly decreased in SFL mice, while the abundance of *Desulfovibrio, unclassified-f-Lachnospiraceae* and *Lachnospiraceae NK4A136-group* was significantly increased. In NASH mice, the abundance of intestinal *Faecalibaculum, Akkermansia* and *Bifidobacterium* was significantly decreased, while the abundance of *Bacteroides, Lachnospiraceae NK4A136-group, unclassified-f-Lachnospiraceae, Blautia, norank-f-Lachnospiraceae, Unclassified-f-Peptostreptococeceae, Alistipes, Enterococcus* and *Ruminiclostridium-9* was significantly increased. In NLF mice, the abundance of *norank-f-Bacteroidales-S24-7-group* was significantly decreased, while the abundance of *Bacteroides, norank-f-Erysipelotrichaceae, Eubacterium-fissiatena-group, Allobaculum, Unclassified-f-Peptostreptococeceae* and *Roseburia* was significantly increased. According to current researches, the above apparently changing bacteria take part in a series of *in vivo* biological processes, such as metabolic and immune regulation. For example, *Akkermansia* has been demonstrated to be involved in regulating metabolic and immune function, protecting mice from HFD induced injuries and may also increase intestinal endocannabinoid levels, thus improving inflammation and intestinal barrier function (Derrien et al., 2017; Zhang et al., 2019). *Allobaculum* was reported to be positively correlated with the expression of angiogenin-like protein 4 (ANGPTL4), a key regulator of lipid metabolism and a circulating mediator of gut microbiota and fat deposition (Zheng et al., 2021). The abundance of intestinal *Blautia* and *Bifidobacterium* was found to be negatively correlated with human visceral fat (Li et al., 2022). Some researches revealed that the consumption of *Blautia* in obese children is related to the deterioration of intestinal inflammation and metabolic phenotype (Benítez-Páez et al., 2020). While other studies found the abundance of *Blautia* and *Allobaculum* was positively correlated with serum triglyceride, low density lipoprotein, IL-6, IL-1β, TNF-α, and body weight, but negatively correlated with serum high density lipoprotein level in HFD-induced fatty liver model (Tang et al., 2018). The *norank-f-Erysipelotrichaceae* is derived from the Erysipelotrichaceae family which has been shown to be associated with gastrointestinal inflammation. While the abundance of *Erysipelotrichaceae* was increased in mice with TNF-α-driven Crohn’s disease-like transmural inflammation, it significantly decreased in patients with recurrent Crohn’s disease. The above different performance may be due to host differences (Dey et al., 2013). *Eubacterium_fissicatena-group*, a butyrate producing bacteria, has been demonstrated to have a high correlation with host obesity and obesity-related metabolic disorder, and the increase of abundance can lead to an increase in the intestinal microbial choline metabolite trimlamine oxide (TMAO), which will affect lipid and hormone homeostasis, enhance the expression of adhesion proteins and induce vascular inflammation. *Roseburia* plays an important role in metabolism and inflammation regulation, and it was confirmed to be efficient in reducing liver steatosis and inflammatory response. In the present study, after analyzing the alteration in the gut microbiota of NAFLD mice with different severity with or without SJP treatment, we found a very interesting phenomenon: all the decreased bacteria were beneficial among the apparent changed SCFAs-producing bacterium; the abundance of bacterium with pro-inflammatory effects, such as *Desulfovibrio*, *Enterococcus* and *Eubacterium_fissicatena-group* was significantly increased; and the abundance of anti-inflammatory bacterium, such as *Akkermansia*, *Lactobacillus*, *norank-f-Bacteroidales-S24-7-group* and *Bifidobacterium* was significantly decreased. Moreover, SJP treatment can effectively increase the intestinal *Roseburia* abundance in SFL mice and the *Akkermansia* abundance in NASH and NLF mice. The beneficial effect of SJP on NAFLD may be associated with the regulation of the above bacterium.

In the present study, the functional prediction of the top 30 highly abundant species in the gut microbiota of NAFLD mice showed that the gut microbiota of SFL and NASH mice was significantly enriched in a series of level3 signal pathways and participated in various biological activities including metabolism, genetic information processing, environmental information processing, cell progression, human disease and organism system. There was no significant difference in the enrichment of gut microbiota in above signal pathways between the NLF mice and the controls. While SJP treatment not only increased the enrichment of gut microbiota in certain pathways in SFL mice, but also increased the enrichment in almost all the level3 pathways involved in NLF mice. Thus, we concluded that SJP may be involved in regulating multiple pathophysiological processes in HFD induced NAFLD mice model by regulating the gut microbiota. However, due to the limited understanding of the function of intestinal microflora and their specific role *in vivo*, the specific mechanism of how their changes affect the occurrence and development of NAFLD, and how the SJP regulates the intestinal microbiota still needs further study.

Current researches has demonstrated that SJP is benefit for NAFLD, but it is not clear whether it has a role in different severity and how it affects the severity of the disease. So we designed this study to further explore the effect of SJP on NASH and NLF based on our previous finding that SJP can improve HFD induced SFL through regulating the intestinal microbiota. In our study, unlike SFL mice models induced by HFD feeding, NASH and NLF mice models are induced by a special HFD feeding CDAHFD because even prolonged HFD feeding is difficult to establish the characteristic inflammation and fibrosis manifestations of the NASH and NLF models. Fortunately, CDAHFD feeding is currently a very mature method to establish NASH and NLF models, which can well simulate the histopathological changes of NASH and NLF. And future models that can better mimic NAFLD may allow for better validation of our current findings.

In summary, we found significant differences in the intestinal microbiota of NAFLD mice with different severity, and SJP could regulate the gut microbiota composition and thereby affect the related biological activities. SJP was much suitable for early treatment of NAFLD as the protective effect diminished with the disease severity and the potential mechanism might be related to the regulation of intestinal microbiota.

## Data availability statement

The original contributions presented in the study are included in the article/Supplementary material, further inquiries can be directed to the corresponding author.

## Ethics statement

The animal study was approved by Experimental Animal Ethics Committee, West China Hospital, Sichuan University. The study was conducted in accordance with the local legislation and institutional requirements.

## Author contributions

PZ: Writing – original draft, Data curation, Formal analysis. JL: Writing – review & editing, Conceptualization, Formal analysis, Funding acquisition, Investigation, Methodology. YM: Writing – original draft, Data curation, Investigation. XZ: Writing – original draft, Formal analysis, Software. LZ: Writing – original draft, Investigation, Methodology. JY: Writing – original draft, Data curation, Methodology. MW: Writing – review & editing, Conceptualization, Supervision. WT: Conceptualization, Funding acquisition, Methodology, Supervision, Writing – review & editing.

## Glossary

ALT: alanine aminotransferase
AST: aspartate aminotransferase
CDAHFD: choline-deficient, L-amino acid-defined high-fat diet
FASN: fatty acid synthase
H&E: haematoxylin-eosin staining
Lxr: liver X receptor
NAFLD: nonalcoholic fatty liver
NASH: non-alcoholic steatohepatitis
SCFAs: short-chain fatty acids
SFL: simple fatty liver
SJP: Sheng-Jiang Powder
Srebp1c: sterol regulatory element binding protein 1C
TC: total cholesterol
TCM: Traditional Chinese medicine
TG: triglyceride
Pparγ: peroxisome proliferator activated receptor γ
