# Supplementary material for: Sheng-Jiang powder ameliorates NAFLD via regulating intestinal microbiota in mice
Source: Front Microbiol. 2024 May 27;15:1387401. doi: 10.3389/fmicb.2024.1387401 (PMC11163104; doi:10.3389/fmicb.2024.1387401)
Supplement: Supplementary file 1 [file Table_1.DOCX]

## Supplementary Table

**Table S1:** Sequences of RT-qPCR primers

| Species | Gene | Forward (5’-3’) | Reverse (5’-3’) |
| --- | --- | --- | --- |
| Mouse | SREBP1c | CTTTGGCCTCGCTTTTCGG | TGGGTCCAATTAGAGCCATCTC |
| Mouse | PPARγ | CTCCAAGAATACCAAAGTGCGA | GCCTGATGCTTTATCCCCACA |

**Table S2.** Effects on serum TC, TG, and body weight following SJP treatment.

| Group | Body weight (g) | P value | TG (mmol/L) | P value | TC (mmol/L) | P value |
| --- | --- | --- | --- | --- | --- | --- |
| BC (n=7) | 25.6±1.71 | — | 1.11±0.17 | — | 3.06±0.12 | — |
| BT (n=7) | 18.7±1.67^***^ | <0.001 | 0.62±0.05^**^ | 0.0098 | 0.96±0.06^***^ | <0.001 |
| BS (n=7) | 17.0±1.54 | 0.1130 | 0.63±0.05 | 0.0666 | 0.63±0.05 | 0.5046 |
| CC (n=7) | 27.16±1.79 | — | 1.04±0.26 | — | 3.36±0.37 | — |
| CT (n=7) | 19.9±2.27^###^ | <0.001 | 0.78±0.17^#^ | 0.0177 | 0.85±0.2^###^ | <0.001 |
| CS (n=7) | 18.96±0.43 | 0.3847 | 0.95±0.16^&^ | 0.0479 | 1.17±0.35^&^ | 0.0266 |

***P* < 0.01 and ****P* < 0.001 vs. BC; ^#^*P* < 0.05 and ^###^*P* < 0.001 vs. CC; ^&^*P* < 0.05 vs. CT. BC: non-alcoholic steatohepatitis control group; BT: non-alcoholic steatohepatitis group; BS: non-alcoholic steatohepatitis+SJP treatment group. CC: liver cirrhosis control group; CT: liver cirrhosis group; CS: liver cirrhosis + SJP treatment group.

## Supplementary Figure


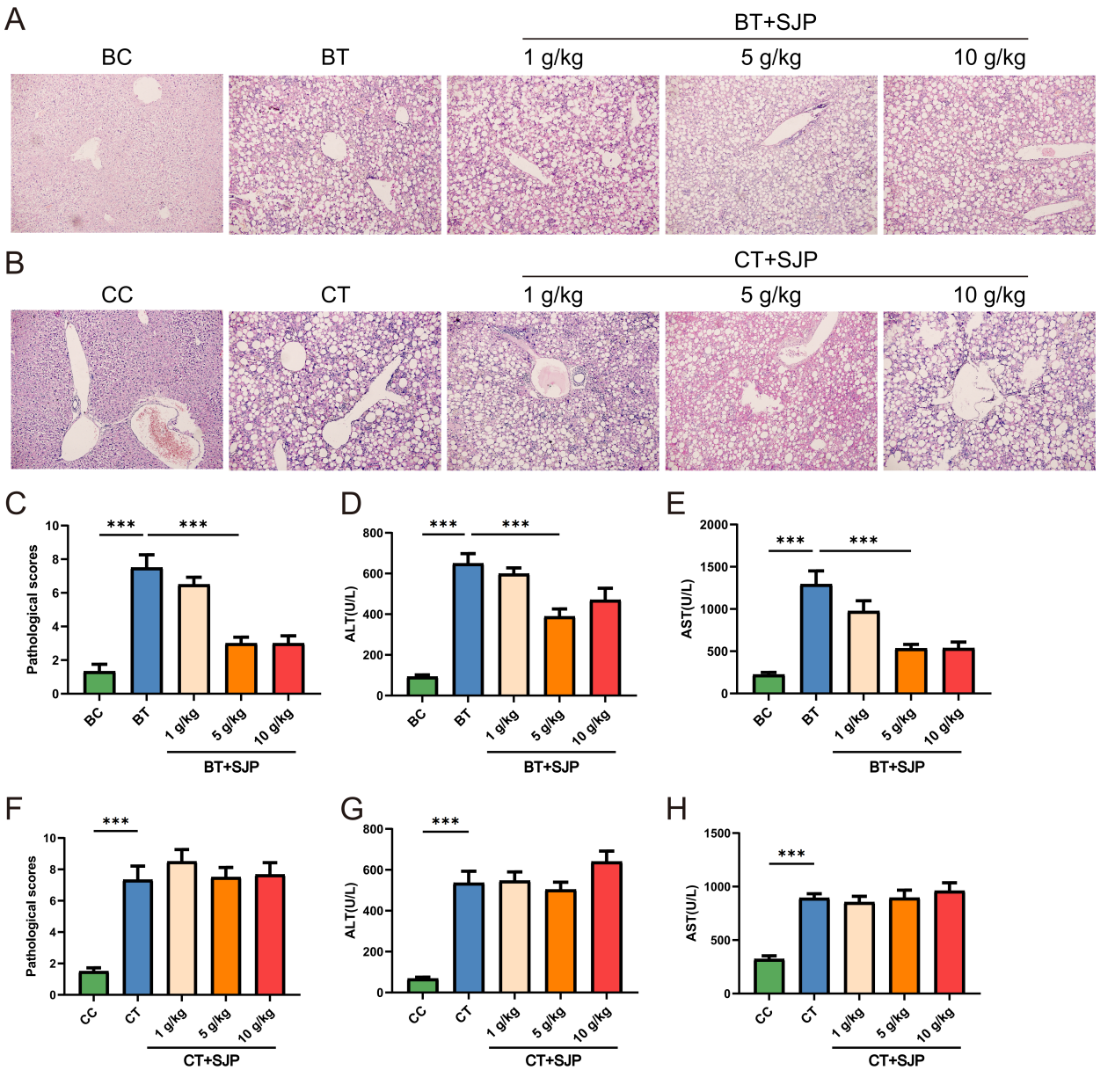


**Fig. S1.** To evaluate the efficacy of different doses of SJP on liver injury. Representative pathological pictures (A–B) and pathological scores (C) of liver tissues. (D–E) The levels of serum ALT and AST. ****P* < 0.001. BC: non-alcoholic steatohepatitis control group; BT: non-alcoholic steatohepatitis group;CC: liver cirrhosis control group; CT: liver cirrhosis group.


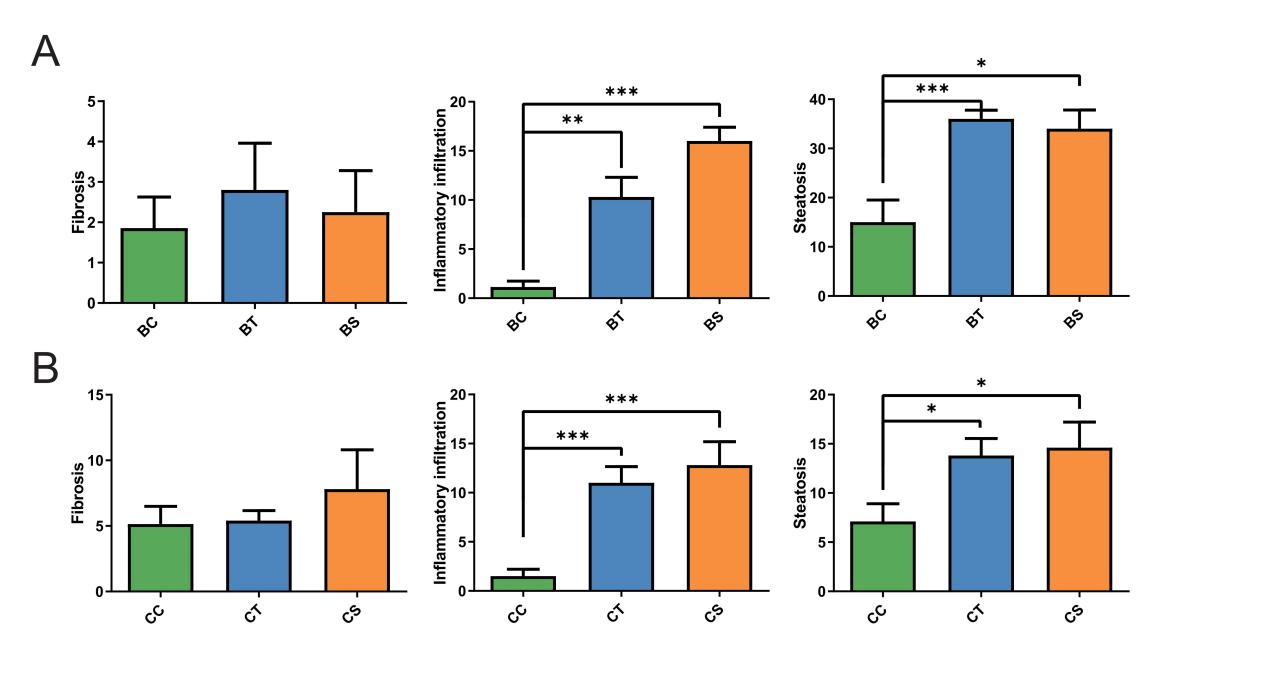


**Fig.** **S2.** The statistical analysis of pathological score. (A) Liver fibrosis, inflammatory infiltration, and steatosis scores in the BC, BT, and BS groups. (B) Liver fibrosis, inflammatory infiltration, and steatosis scores in the CC, CT, and CS groups. **P* < 0.05, ***P* < 0.01, and ****P* < 0.001. BC: non-alcoholic steatohepatitis control group; BT: non-alcoholic steatohepatitis group; BS: non-alcoholic steatohepatitis+SJP treatment group. CC: liver cirrhosis control group; CT: liver cirrhosis group; CS: liver cirrhosis + SJP treatment group.


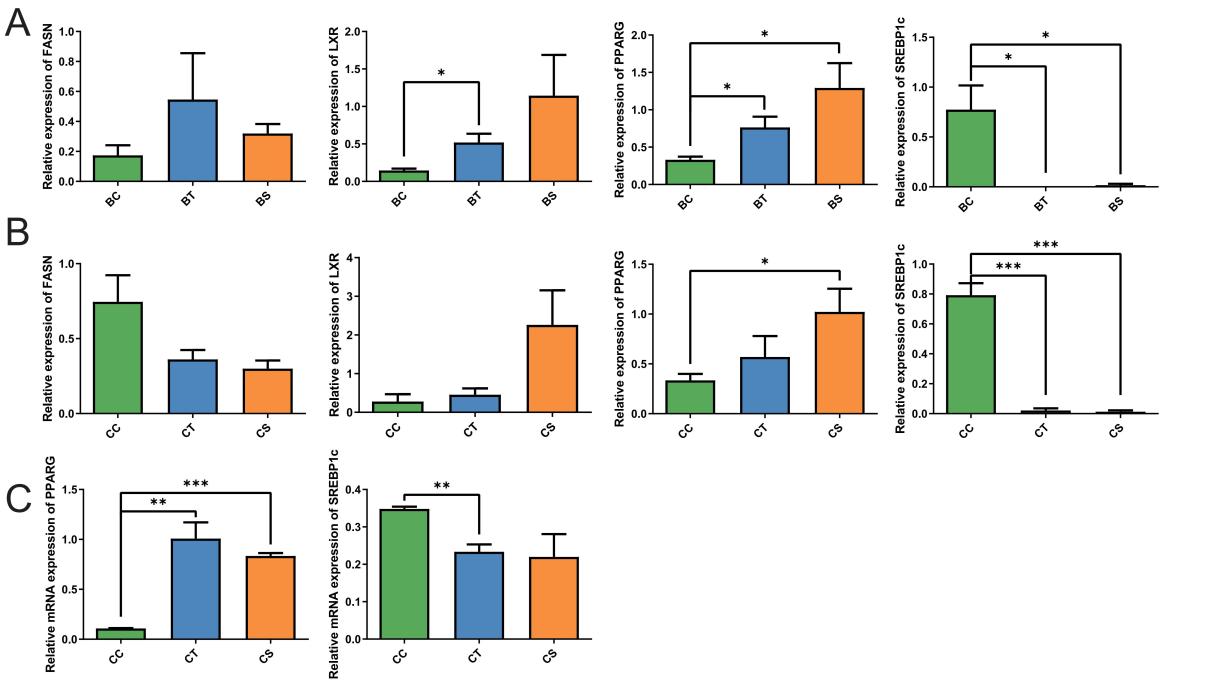


**Fig. S3.** Statistical analysis of the qPCR and Western blotting results. (A) Western blot analysis of protein expression of Lxr, Pparγ, Srebp1c and Fasn in BC, BT and BS groups. (B) Western blot analysis of protein expression of Lxr, Pparγ, Srebp1c and Fasn in CC, CT and CS groups. (C) qPCR analysis of gene expression of Pparγ and Srebp1c in CC, CT and CS groups. **P* < 0.05, ***P* < 0.01 and ****P* < 0.001. BC: non-alcoholic steatohepatitis control group; BT: non-alcoholic steatohepatitis group; BS: non-alcoholic steatohepatitis+SJP treatment group. CC: liver cirrhosis control group; CT: liver cirrhosis group; CS: liver cirrhosis + SJP treatment group.
