# Supplementary figures and images for: Sheng-Jiang powder ameliorates NAFLD via regulating intestinal microbiota in mice
Source: Front Microbiol. 2024 May 27;15:1387401. doi: 10.3389/fmicb.2024.1387401 (PMC11163104; doi:10.3389/fmicb.2024.1387401)

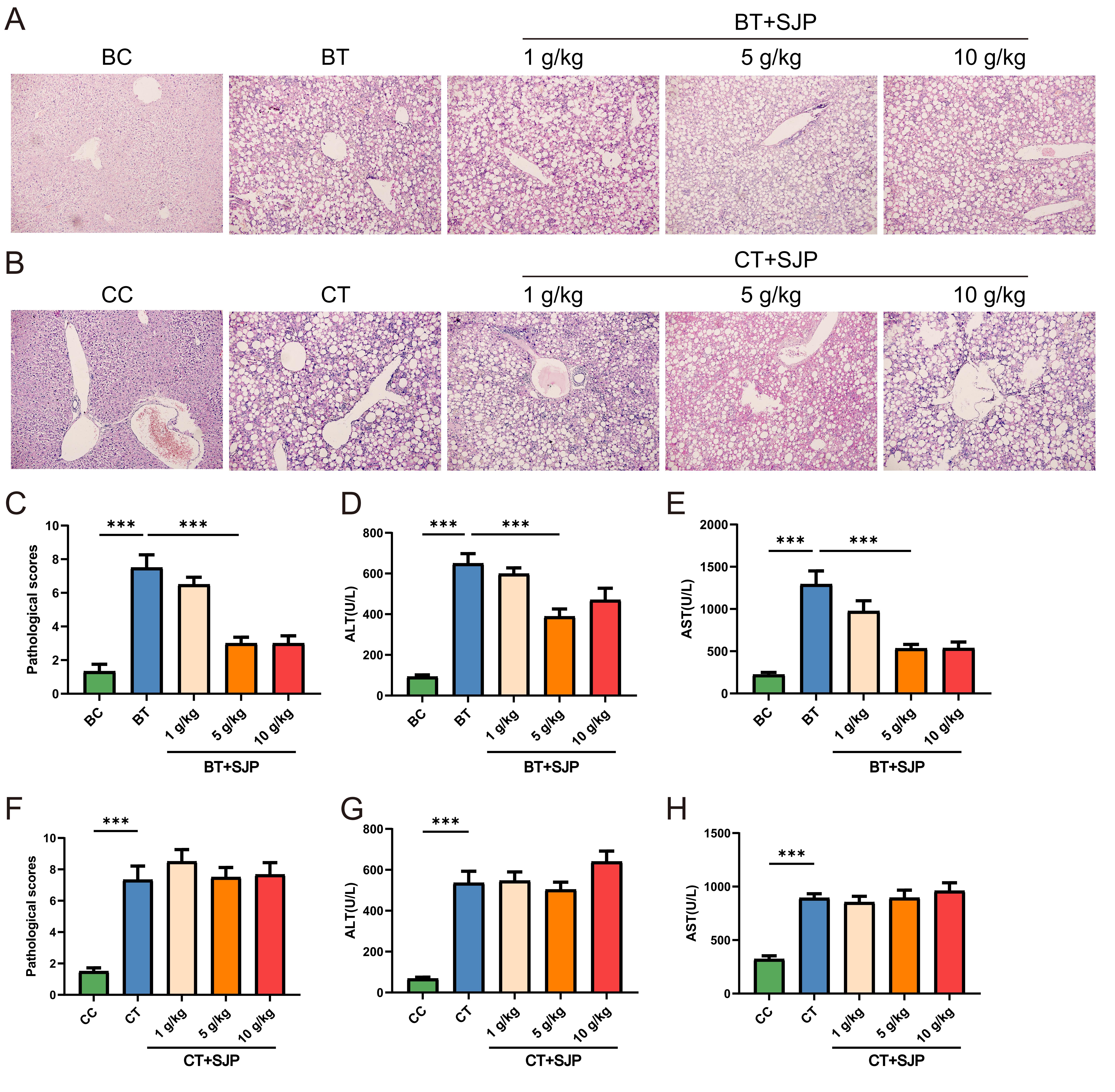

Supplement: Supplementary file 2 [file Image_1.JPEG]

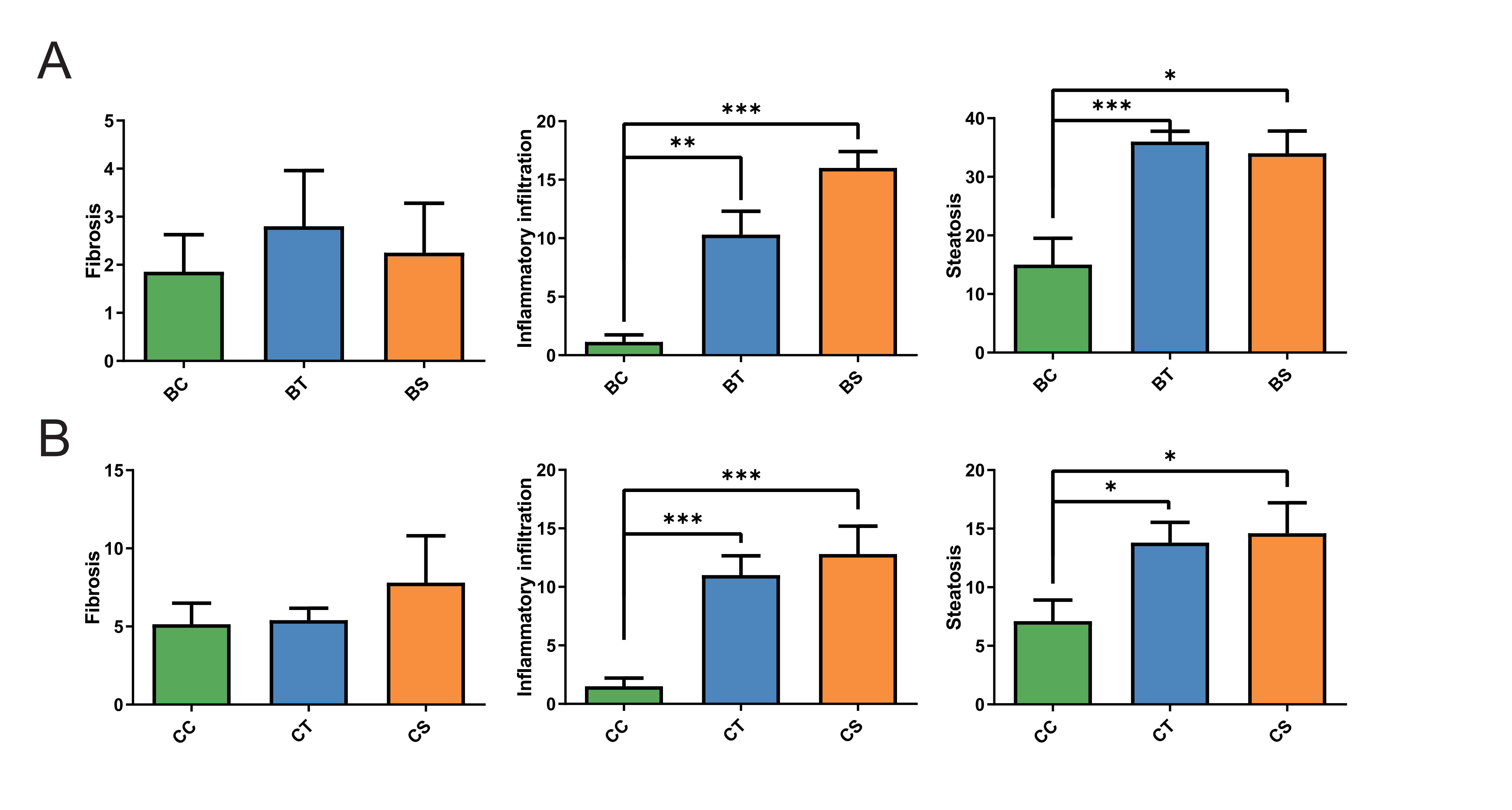

Supplement: Supplementary file 3 [file Image_2.JPEG]

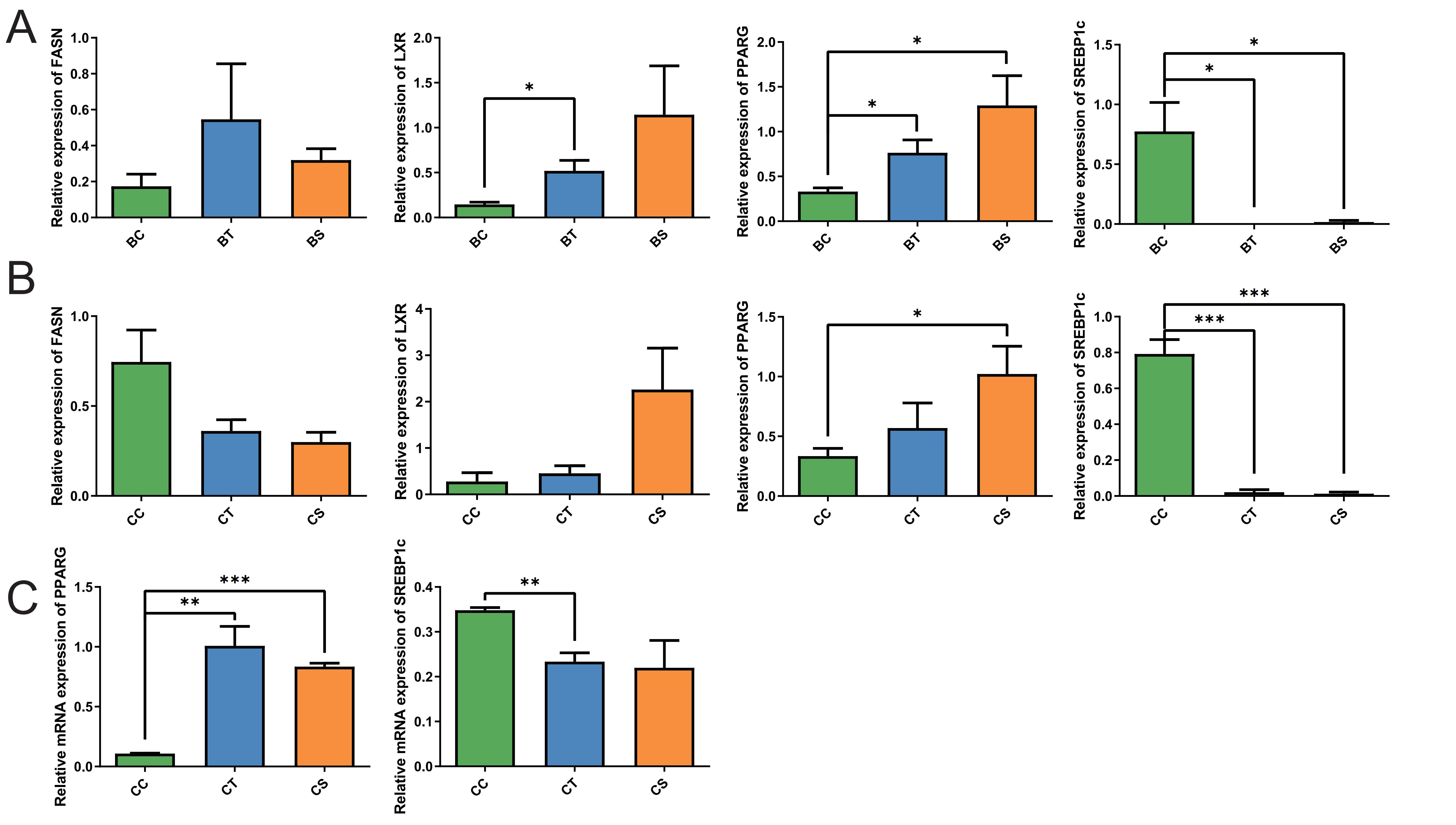

Supplement: Supplementary file 4 [file Image_3.JPEG]
